# Treatment with TNF-α or bacterial lipopolysaccharide attenuates endocardial endothelial cell-mediated stimulation of cardiac fibroblasts

**DOI:** 10.1186/1423-0127-16-21

**Published:** 2009-02-17

**Authors:** Leena Kuruvilla, Cheranellore Chandrasekharan Kartha

**Affiliations:** 1 Division of Cellular & Molecular Cardiology, Sree Chitra Tirunal Institute for Medical Sciences and Technology, Thiruvananthapuram 695011, India; 2 Professor of Eminence, Disease Biology and Molecular Medicine, Rajiv Gandhi Center for Biotechnology, Thiruvananthapuram 695014, India

## Abstract

**Background:**

The endocardial endothelium that lines the inner cavity of the heart is distinct from the microvascular endothelial cells and modulates cardiac muscle performance in a manner similar to the vascular endothelial modulation of vascular structure and vasomotor tone. Although the modulatory effects of endocardial endothelium (EE) on cardiomyocytes are firmly established, the regulatory effects of endocardial endothelium on the cardiac interstitium and its cellular components remain ill defined.

**Methods and Results:**

We investigated whether the stimulatory effect of EE on cardiac fibroblasts would be altered when EECs are activated by the cytokine tumor necrosis factor-α (TNF-α) or the endotoxin bacterial lipopolysaccharide (LPS). Both TNF-α and LPS were found to independently attenuate the stimulatory effect of EE on cardiac fibroblasts. These agents lowered the synthesis or release of ET-1 and increased the secretion of TGF-β and NO.

**Conclusion:**

The findings of this study using endocardial endothelial cells (EECs) and neonatal cardiac fibroblasts demonstrate that pro-inflammatory cytokines cause altered secretion of paracrine factors by EECs and inhibit proliferation and lower collagen synthesis in fibroblasts. These changes may influence fibroblast response and extra cellular matrix remodeling in pathological conditions of the heart.

## Background

The endocardial endothelium (EE) that lines the inner cavity of the heart is distinct from the microvascular endothelial cells in terms of embryological origin, cytoskeletal organization, receptor – mediated functions, electrophysiological properties, release of prostanoids and growth characteristics in culture [1]. The EE is strategically situated between the circulating blood and the cardiac muscle and it modulates cardiac muscle performance exactly as the vascular endothelium modulates vascular structure and vasomotor tone. Brutsaert *et al *[2] have demonstrated that EE is an important modulator of subjacent cardiac muscle performance. Dysfunction of this interface could be a critical factor in various pathological conditions of the heart. Evidence of physiologically significant paracrine interactions between the endocardial cell populations and muscle cells of the heart accrued from studies on factors of endothelial origin such as endothelins, angiotensin II, nitric oxide (NO), natriuretic peptides, bradykinin prostaglandins, adenylpurines, myofilament desensitizing element and enzymes such as angiotensin converting enzyme and kininase. Importantly, an imbalance in the turnover of these factors in cardiovascular diseases may potentially promote alterations in the extra cellular matrix (ECM) and disturb cardiomyocyte function. In fact, heart function is reported to be significantly affected by increased cytokine production in a setting of endotoxic shock, transplant rejection and ischemia/reperfusion [3]. Endothelium derived factors such as TNF-α, IL-1β, IL-6 and TGF-β may exert autocrine and paracrine effects on fibroblast growth and collagen turnover as well.

Whereas the modulatory influence of EE on cardiomyocytes is well established, the effects of EE on the cardiac interstitium and its cellular components particularly the fibroblasts, which maintain the extracellular matrix homeostasis are far less defined [4]. In this regard, we had recently reported a significant increase in cardiac fibroblast proliferation and collagen synthesis when the cells are grown in EEC conditioned media [5]. In the present study, we investigated whether the stimulatory effect of EE on cardiac fibroblasts would be altered in conditions where TNF-α or the endotoxin bacterial LPS activates EECs. Our results suggest that TNF-α or LPS – activated EE cells attenuate proliferation and collagen synthesis in cardiac fibroblasts.

## Materials and methods

Tissue culture media and all supplements were procured from Sigma-Aldrich, St. Louis, USA. [^3^H]-Thymidine and [^3^H]-Proline was obtained from Board of Radioactivity and Isotope Technology (BRIT), Mumbai, India. DiI-Acetylated LDL was purchased from Molecular Probes, Netherlands.

### Preparation of cultures of endocardial endothelial cells

Endocardial endothelial cells were isolated from freshly collected pig hearts by the method previously described by Smith *et al *[6]. Briefly, the ventricles were filled with 0.1% collagenase (Type IA) in medium E199 and incubated for 45 minutes. The released cells were resuspended in complete medium (medium E199 supplemented with 20% fetal bovine serum (FBS), 1% endothelial cell growth factor, 100 U/ml benzyl penicillin and 100 μg/ml streptomycin) and seeded in gelatin – coated culture dishes. Confluent cultures were sub – cultured using 0.025% trypsin – 0.02% EDTA. The cells were identified as endothelial cells by their cobblestone appearance, positive staining for factor VIII antigen and incorporation of DiI-acetylated low-density lipoproteins.

### Preparation of cultures of cardiac fibroblasts

Cardiac fibroblasts were isolated from 3- to 4-day-old Wistar rat pups. The heart tissue was minced and digested with 0.03 % collagenase and 0.03 % trypsin. The supernatants were centrifuged and cell pellet was resuspended in medium M199 with 10% FBS. Cardiac fibroblasts attached within 90 minutes. The cells were grown to confluence and passaged with 0.025% trypsin-0.02% EDTA mixture. The cells were identified as fibroblasts by their spindle morphology, positive staining for vimentin and negative staining for factor VIII antigen.

All experiments had the approval of the Institutional Animal Ethics Committee.

### Preparation of conditioned medium

The experiments were performed on EECs from the 3^rd ^– 4^th ^passages. Cells were seeded at a density of 2 × 10^5 ^cells/ml in 35 mm culture dishes. At confluence, the cells were made quiescent by reducing the serum content of the medium to 0.4% for 24 hours. On the day of the experiment, the cells were washed with phosphate – buffered saline and treated with either 10 ng/ml TNF-α or 1 μg/ml bacterial LPS. Cells incubated in 0.4% FBS containing medium served as control. At the end of the incubation period of 24 hours, the medium was collected, centrifuged to remove cellular debris and stored at -80°C until use. Dulbecco's modified Eagle's medium (DMEM) was used for generating conditioned medium for the collagen synthesis experiments.

### Proliferation assay

DNA synthesis in cardiac fibroblasts was measured in terms of [^3^H]-Thymidine incorporation into DNA (n = 15) as described earlier [7]. Cells were seeded at a density of 1 × 10^5 ^cells/ml and incubated for 24 hours. Cells from passage 2 were made quiescent by incubating them in medium containing 0.4% FBS for 24 hours. Following incubation of the cells with [^3^H]-Thymidine at a final concentration of 1 μCi/ml for 24 hours, acid – insoluble radioactivity was determined by using liquid scintillation spectrometry.

### Collagen synthesis assay

Incorporation of [^3^H]-Proline by cardiac fibroblasts was taken as a measure of collagen synthesis (n = 9). Cardiac fibroblasts at confluence were incubated in DMEM supplemented with 0.4% FBS, 24 hours prior to treatment. Subsequently, cells were incubated for 24 hours in either 0.4% FBS or EEC – conditioned medium, containing 2 μCi/ml [^3^H]-Proline and 50 μg/ml ascorbic acid in DMEM. The supernatant was collected and cells were lysed using 1% Triton-X 100 containing 5 mM N-ethylmaleimide. The cell lysate and medium were pooled and the mixture divided into two aliquots. Proteins in one aliquot were precipitated with 10% ice-cold TCA. The second aliquot was digested with 30 μg/ml collagenase Type VII in Tris-CaCl_2 _buffer (pH – 7.4) for 5 hours at 37°C. At the end of collagenase digestion, the proteins were precipitated with 10% ice-cold TCA. The TCA-precipitated material in the two aliquots was filtered separately onto Whatman no.3 filter paper. Radioactivity was determined using a scintillation counter. Collagen synthesis was calculated using the equation:

$$\text{Collagen (\% of total protein)}=\frac{\text{Collagenase released cpm }\times \;100}{(\text{Non-collagen cpm }\times \text{ f})\;+\text{ collagenase released cpm}}$$

A correction factor of f = 5.4 for non-collagen protein was used to adjust for the relative abundance of proline and hydroxyproline in collagen containing proteins [8].

### Measurement of EEC – derived factors in the conditioned medium

The conditioned medium was assayed (n = 6) for the levels of EEC – derived factors, ET-1 (Cayman Chemical, MI, USA), Ang II (SPI BIO, France) and TGF-β1 (Biosource International, Belgium) by ELISA using commercially available kits.

### Statistics

Sample means were compared using Student's t-test. ANOVA was employed for group comparisons where there were more than two groups. The values are expressed as mean ± SD. p < 0.05 was considered statistically significant.

## Results

### Effect of TNF-α and LPS on EEC-induced proliferation of cardiac fibroblasts

**Figure 1:**
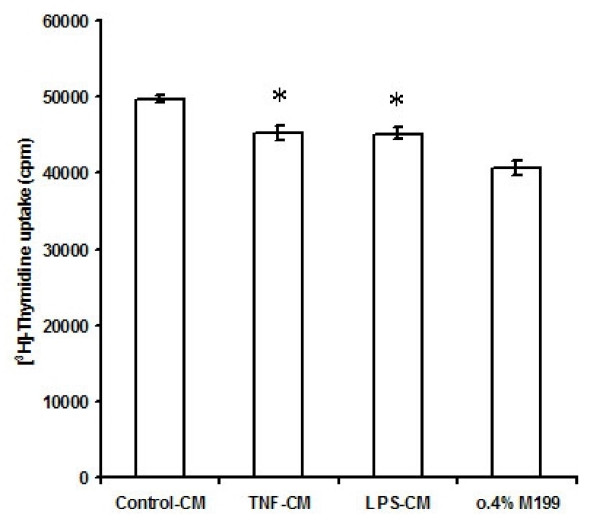
**(^3^H)-Thymidine uptake by cardiac fibroblasts incubated with conditioned medium from EECs treated with TNF-α and LPS**. The values are mean ± SD (n = 15; Control-CM vs TNF-CM & Control-CM vs LPS-CM, *p < 0.01. Control-CM vs. 0.4% M199, *p < 0.01). Control-CM = Fibroblasts grown in conditioned medium from untreated endocardial endothelial cells, TNF-CM = Fibroblasts grown in conditioned medium from endocardial endothelial cells treated with TNF-α, LPS-CM = Fibroblasts grown in conditioned medium from endocardial endothelial cells treated with LPS, 0.4% M199 = Fibroblasts grown in M199 containing 0.4% FBS. 'n' is the number of times each experiment was done.

On incubation with EE – conditioned medium, [^3^H]-Thymidine incorporation into cardiac fibroblast DNA increased by 22%. The stimulatory effect was however attenuated when cardiac fibroblasts were incubated in medium conditioned by TNF-α or LPS treated EECs. [^3^H]-Thymidine counts per minute (cpm) with medium conditioned by untreated EECs was 49678 ± 418.7. [^3^H]-Thymidine counts per minute (cpm) with medium conditioned by EECs treated with TNF-α or LPS were 45271.7 ± 1008.7 cpm and 45163.6 ± 802.5 cpm respectively. The basal [^3^H]-Thymidine counts per minute (cpm) with 0.4% M199 was 40676 ± 985 cpm. Thus, TNF-α treated EECs attenuated [^3^H]-Thymidine incorporation into DNA of cardiac fibroblasts by 11.3 % (n = 15; p < 0.01) and LPS treated EECs attenuated [^3^H]-Thymidine incorporation into DNA of cardiac fibroblasts by 11% (n = 15; p < 0.01) (Figure 1).

### Effect of TNF-α and LPS on EEC-induced collagen synthesis by cardiac fibroblasts

**Figure 2:**
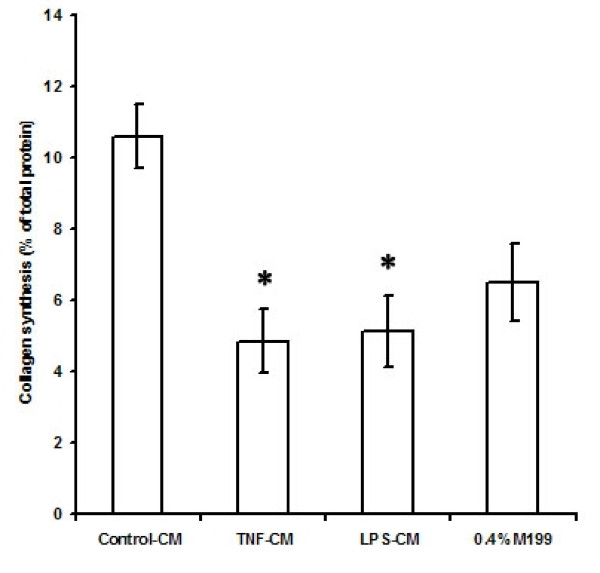
**Collagen synthesis by cardiac fibroblasts in response to incubation with conditioned medium from endocardial endothelial cells treated with TNF-α and LPS for 24 hours**. The values are mean ± SD (n = 6; Control-CM vs TNF-CM and LPS-CM, *p < 0.05. Control-CM vs 0.4% M199 *p < 0.05). Control-CM = Fibroblasts grown in conditioned medium from untreated endocardial endothelial cells, TNF-CM = Fibroblasts grown in conditioned medium from endocardial endothelial cells treated with TNF-α, LPS-CM = Fibroblasts grown in conditioned medium from endocardial endothelial cells treated with LPS, 0.4% M199 = Fibroblasts grown in M199 containing 0.4% FBS. 'n' is the number of times each experiment was done.

The effect of medium conditioned by TNF-α or LPS treated EECs, on collagen synthesis by cardiac fibroblasts is shown in Figure 2. The basal rate of collagen synthesis by cardiac fibroblasts in 0.4% M199 was 6.51 ± 1.0 % of the total protien and by fibroblasts grown in medium conditioned by untreated EECs was 10.6 ± 0.9 % of the total protien. Collagen synthesis by cardiac fibroblasts was reduced to 4.85 ± 0.9 % and 5.12 ± 1 % of the total protein respectively when the cells were grown in medium conditioned by TNF-α or LPS treated EECs (n = 6; Control-CM vs TNF-CM and LPS-CM, p < 0.05). Whereas a 6 % increase in collagen synthesis was seen in fibroblasts grown in medium conditioned by untreated EECs, rate of collagen synthesis in cardiac fibroblasts incubated with conditioned medium from EECs treated with TNF-α or LPS was seen to decrease by 25% and 21% respectively, compared to the control (Figure 2).

### Assay of endocardial endothelial cell released factors in the culture supernatants

Levels of endothelium-derived factors ET-1, AII, and TGF-β1, were determined by ELISA according to the manufacturer's protocols. NO released into the medium was measured as nitrite by Griess reaction.

**Figure 3:**
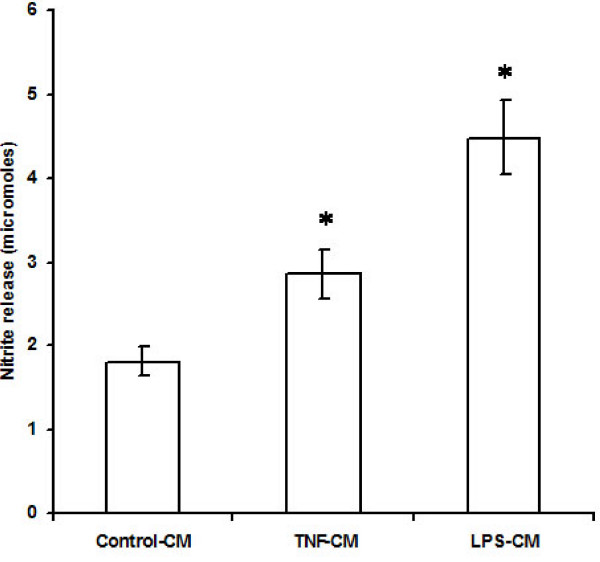
**Nitrite release by endocardial endothelial cells in response to proinflammatory agents TNF-α and LPS**. The values are mean ± SD (n = 6;*p < 0.05). Control-CM = Conditioned medium from untreated endocardial endothelial cells, TNF-CM = Conditioned medium from endocardial endothelial cells treated with TNF-α, LPS-CM = Conditioned medium from endocardial endothelial cells treated with LPS. 'n' is the number of times each experiment was done.

Treatment of EECs with TNF-α and LPS caused increased release of nitrite from the cells into the culture supernatant. The amount of nitrite released into the medium conditioned by untreated EECs was 1.81 ± 0.17 μM. TNF-α or LPS treatment of EECs increased nitrite levels in the culture supernatant to 2.86 ± 0.29 μM and 4.48 ± 0.44 μM respectively (n = 6; p < 0.05). Thus, stimulation of EECs with TNF-α or LPS increased nitrite release from EECs by 58% and 147% respectively (Figure 3).

ET-1 levels and TGF-β levels in the culture supernatant of EECs treated with TNF-α were 5.56 ± 0.02 ng/ml and 7.21 ± 0.1 ng/ml respectively compared to ET -1 and TGF-β levels of 6.6 ± 0.01 ng/ml and 6.56 ± 0.05 ng/ml respectively in the culture supernatant of untreated EECs (n = 6; p < 0.01). Thus, TNF-α depressed the release of ET-1 by 16% but increased TGF-β release by 10% from EECs.

**Figure 4:**
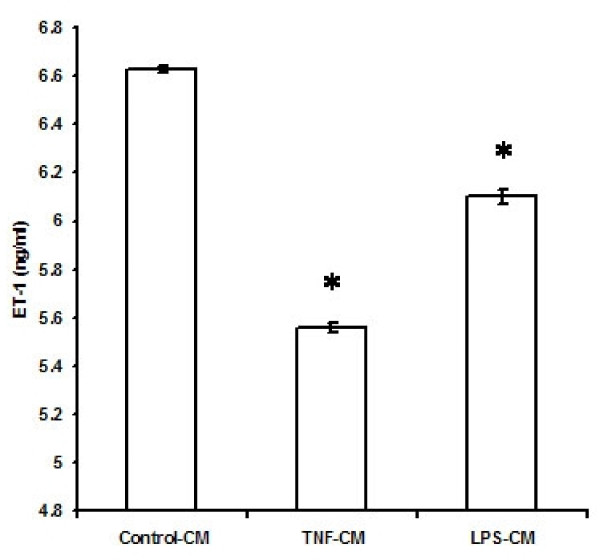
**Effect of TNF-α and LPS on ET-1 release by endocardial endothelial cells**. The values are mean ± SD (n = 6;*p < 0.01). Control-CM = Conditioned medium from untreated endocardial endothelial cells, TNF-CM = Conditioned medium from endocardial endothelial cells treated with TNF-α, LPS-CM = Conditioned medium from endocardial endothelial cells treated with LPS. 'n' is the number of times each experiment was done.

**Figure 5:**
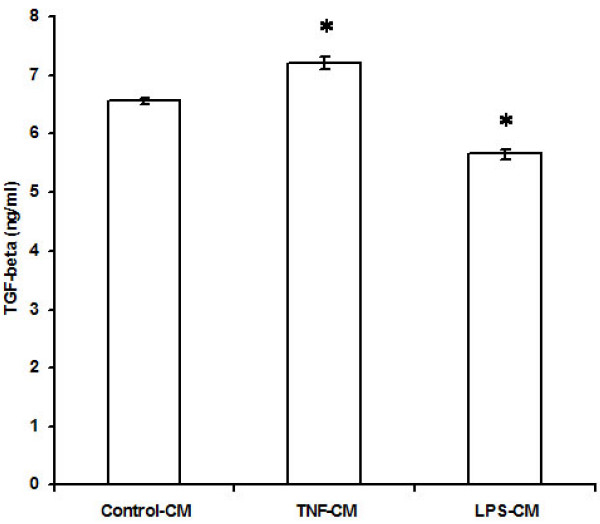
**Effect of TNF-α and LPS on TGF-β release by endocardial endothelial cells**. The values are mean ± SD (n = 6;*p < 0.01). Control-CM = Conditioned medium from untreated endocardial endothelial cells, TNF-CM = Conditioned medium from endocardial endothelial cells treated with TNF-α, LPS-CM = Conditioned medium from endocardial endothelial cells treated with LPS. 'n' is the number of times each experiment was done.

When EECs were treated with LPS, the ET-1 levels in the culture supernatant was 6.1 ± 0.03 ng/ml and TGF-β levels were 5.66 ± 0.08 ng/ml compared to the ET-1 levels of 6.63 ± 0.01 ng/ml and TGF-β levels of 6.56 ± 0.05 ng/ml in the culture supernatant of untreated EECs (n = 6; p < 0.01). Thus, treatment of EECs with LPS reduced ET-1 secretion by 8% and TGF-β secretion by 13.7% (Figures 4 and 5).

Angiotensin II levels were undetectable in all the culture supernatants.

## Discussion

It is well recognized that vascular endothelial cells exert marked regulatory influence on subjacent non-endothelial cells such as myocytes, fibroblasts, pericytes and smooth muscle cells (SMCs) [9-13]. Given the regional differences in the functional properties of the endothelium, we had earlier investigated the role of EE in modulating cardiac fibroblast proliferation and collagen synthesis and found that EE has a stimulatory effect on cardiac fibroblasts [5]. In the present study we explored whether pro – inflammatory agents such as TNF-α and LPS modulate this stimulatory effect. Interestingly, both TNF-α and LPS were found to attenuate the stimulatory effect of EE on cardiac fibroblasts.

The inflammatory effects of TNF-α and LPS on vascular endothelial cells are well – characterized. Endothelial cells obtained from different sites exhibit varied responses to cytokines and LPS [14]. However, the effects of TNF-α and LPS on endocardial endothelial cells have not been previously reported.

In the present study, [^3^H]-Thymidine incorporation as well as rate of collagen synthesis were significantly lower in cardiac fibroblasts grown in conditioned medium from EECs treated with either TNF-α or LPS, when compared to the cells grown in EEC conditioned medium and not treated with either agents. Neither TNF-α nor LPS affected the viability of the cells. Concentrations of TNF-α up to 1000 ng/ml have been reported to cause reduction in collagen synthesis in cardiac fibroblasts without affecting the cell numbers [15]. Our study demonstrated not only the direct inhibitory action of TNF-α on collagen synthesis in cardiac fibroblasts, but also its ability to attenuate the stimulatory effect of EECs on collagen production by cardiac fibroblasts. Yokoyama et al [16] proposed that TNF-α could act as an autocrine/paracrine mediator in myocardial remodeling. The cytokine increases both the expression and activity of matrix metalloproteinases (MMPs) which regulate matrix turnover [17]. In studies investigating the direct effect of TNF-α on cardiac fibroblasts, it has been observed that the cytokine decreases total collagen synthesis [18,19].

We also observed alterations in the release of endothelium-derived factors, such as NO, TGF-β and ET-1 into the conditioned medium when EECs were treated with TNF-a or LPS, which in turn could contribute to the altered response elicited in cardiac fibroblasts treated with the conditioned medium. EECs released significantly higher levels of nitrite in response to the pro-inflammatory agents. A notable action of TNF-α is its ability to induce nitric oxide synthase (NOS) activity in different cell types, including endocardial cells [6,20]. Nitric oxide (NO) is an important modulator of TNF-α in the heart. In addition to being an immunomodulator and a potent inhibitor of platelet aggregation and cell migration, NO is also an anti-mitogen [21-23]. In a study using endothelial cells and SMCs from coronary arteries, inhibition of NO has been shown to cause an increase in the concentration of collagen types I and III. The data also supports an inhibitory role for NO on collagen synthesis [24]. Extrapolating these findings to the present study, it is tempting to postulate that the attenuation of the proliferative response in cardiac fibroblasts is the result of increased release of NO from EECs on treatment with TNF-α or LPS.

Levels of endothelin (ET-1) in the conditioned medium from EECs treated with TNF-∝ or LPS were found to be lower, raising the possibility that a decrease in the levels of ET-1 may contribute to the diminished proliferative response in fibroblasts. Our earlier studies using endothelin inhibitors had shown that the stimulatory effect of EECs on cardiac fibroblasts is mediated by ET-1 [5].

Treatment of EECs with TNF-∝ also resulted in an elevation of TGF-β in the conditioned medium. Dose-dependent increase in TGF-β release has been observed in microglial cells treated with TNF-∝ [25]. It is possible that TNF-∝ may regulate TGF-β expression as a feed back mechanism to limit extra cellular degradation in response to injury. TGF-β being inhibitory to cardiac fibroblast proliferation, the increased levels of this peptide in the conditioned medium from TNF-∝ treated EECs obviously accentuates the attenuation of proliferation brought about by increased NO and decreased ET-1.

LPS causes cardiac dysfunction by enhancing cardiac-derived inflammatory mediator expression, associated with the release of pro-inflammatory cytokines such as TNF-α and IL-1β and over production of NO [26,27]. Human coronary endothelial cells stimulated with LPS express higher levels of TNF mRNA and release increased levels of the cytokine [28]. Since most of the effects of LPS are through TNF, it can be assumed that in cardiac fibroblasts, the endotoxin elicits responses similar to that induced by TNF-α.

In this study, we have explored the independent effects of TNF-α and LPS on fibroblast function mediated through major mediators released by EECs. It is possible that other EE-derived mediators are also involved in the modulation of fibroblast growth and collagen synthesis. A limitation of the study is the usage of cells from two different species, but in previous studies on the interaction of endothelial cells with their neighboring cells, others have also employed cells derived from different species [12].

## Conclusion

This study suggests that pro-inflammatory cytokines can cause altered expression of paracrine factors in EECs, that in turn may inhibit proliferation and lower collagen synthesis in cardiac fibroblasts. The effects of TNF-α or LPS on EECs as observed in our study may be of significance in pathological conditions such as cardiac failure and during post inflammatory wound healing where different cytokines are up – regulated in the tissues.

## Competing interests

The authors declare that they have no competing interests.

## Authors' contributions

LK participated in the design of the study, carried out the tissue culture experiments, various assays, performed the statistical analysis and drafted the manuscript. CCK conceived of the study, participated in its design, participated in the writing of the manuscript and coordinated the study. Both authors read and approved the final manuscript.
